# Some Conditions Resembling Hip-Joint Disease in Children

**Published:** 1911-10-07

**Authors:** G. H. Edington

**Affiliations:** Assistant Surgeon Western Infirmary, and Extra Honorary Surgeon Royal Hospital for Sick Children, Glasgow.


### HOSPITAL CLINICS.

### Some Conditions Resembling Hip-Joint Disease in Children.

By G. H. EDINGTON, M.D.Glasg., F.R.F.P.S.G., Assistant Surgeon Western Infirmary, and Extra Honorary Surgeon Royal Hospital for Sick Children, Glasgow.

It is not difficult to understand the interest which attaches to all affections of the hip-joint in children. The frequency, at an early age, of tuberculous disease of the joint and the gravity of the prognosis are ever present to the surgeon's mind. It is, therefore, not surprising that cases of limping come frequently under the notice of the general practitioner, or are brought to the clinique of a children's hospital.

Apart from the mere question of correct diagnosis, the grave prognosis attaching to morbus coxæ stimulates the surgeon in his endeavour to ascertain the cause of the disability in all cases of lameness, and he experiences a feeling of relief when he is able to decide that it is of a simple nature. It must be confessed that in some cases the cause, even after repeated examination, remains obscure. The child limps and is wearied after using the limb for a short space of time, and the characteristic signs of morbus coxæ are absent. After a little the patient usually drifts elsewhere and the surgeon remains in doubt as to the true nature of the malady. In a few cases of those which remain under observation, unmistakable signs of tuberculosis of the hip are manifested later. It is not with any of those, however, but with cases which clear up under observation that I propose at present to deal. These cases fall naturally into groups, and I shall mention first those of more common occurrence.

### Acute Traumatic Synovitis.

In the cases which belong to this category there may or may not be a definite history of injury. It may be that in some the occurrence of injury is actually known; but in others there may be nothing more than a presumption that the child has hurt himself while at play. There is limping, which draws attention to the child, and this may be accompanied by tiredness, or by actual pain. Sometimes pain is a very prominent symptom. In this connection the existence of a rheumatic diathesis has to be borne in mind, and may modify the treatment of the case. That any injury occurring in a rheumatic subject may give rise to pain which is not recovered from till anti-rheumatic remedies have been administered has long been well known, and attention has been recently directed to it by Mr. Whitelocke, of Oxford.* Another thing to be kept in mind is the facility with which in some children a habit is contracted. The limping and rigidity remain in such individuals long after one would expect them to have disappeared, and one is driven in these cases to the use of a term which is not so frequently heard now as formerly, viz. "neuromimesis."

**Sprains and Allied Injury of Joints,* second edition, p. 29. London, 1910.

As illustrating these remarks I would take the following from my case-books:—

In September 1902 I saw a little girl, aged about four years, the daughter of a medical colleague. She had been running about at play in her usual health on the previous evening, but on being taken out of bed the next morning she cried and would not put her right foot to the ground. Pain at the knee was complained of. Examination of the knee gave a negative result; but the hip was held in a flexed position, and there was restriction of the movements of the joint. Temperature was normal, and no tenderness about the joint could be elicited.

There was no definite history of injury, and as there was a tendency to rheumatism on the father's side, it was thought advisable to administer anti-rheumatic treatment. Sodium salicylate (gr. ii.) was ordered, to be taken night and morning, and the child kept in bed. Two days later she was almost well and kicking the limb freely about in bed. She was allowed up, the salicylate was stopped, and a tonic substituted. She remained well of the hip condition, but some years later suffered an attack of rheumatic fever. The rapid recovery of this case, notwithstanding the existence of a rheumatic tendency, contrasts with the following:—

### A Typical Case.

On November 29, 1910, I was asked to see the daughter of a medical man, a little girl aged five years, who was suffering from acute pain at the right hip and inability to use the limb. For fourteen days previously she had complained, off and on, of discomfort in the right groin. There was no actual history of injury; but the patient being, as her mother expressed it, a "tomboy," it was presumed that she had strained the part while at play. On the day when I saw her she had screamed with pain when having her boot put on in the morning. She was put back to bed, and when I saw her she was lying supine with the right hip flexed. The joint was held fairly rigid on my attempting to make passive movements; but the patient was able herself to extend the limb almost completely without arching the lumbar spine. There was slight enlargement of inguinal glands on both sides.

I advised the parents that she had probably strained the joint or muscles, and that rest in bed was all that was needed to bring about a cure. Two days later improvement was noted, and although there was no suspicion of rheumatism, salicylate of soda was ordered. On December 3 it was observed that she lay with the limb in the attitude characteristic of the early stage of morbus coxæ—flexion, abduction, and external rotation—and pressure applied to the great trochanter caused pain which was referred to the head of the femur. On December 5 she was evidently much easier and was sitting up in bed. There was, however, some lordosis when she was laid flat. Salicylate was ordered to be continued. On December 10 she was apparently quite well, and the movements of the joint were quite free. She seemed very frightened about herself, however, and refused to put the foot to the ground. She was allowed up on the sofa a few days later and shortly afterwards was running about as freely as ever.

The slow progress of this case towards recovery was a little disconcerting, and I had begun to think that I must revise my diagnosis, in favour of morbus coxæ. From what the child's mother told me, however, I came to the opinion that the patient was making the most of her condition, and I advised that no mention of it should be made in her presence and that she should be allowed to get up as she might wish. This plan proved successful, and in a few days thereafter I was glad to learn that she was getting about and apparently in her usual health. Since then there has been no word of further trouble.

I should add that in this case the possibility of tuberculosis could not at first be altogether excluded, as I had some time previously operated on an elder sister of the patient for tuberculous glands of the neck; but the ultimate course of the hip trouble showed, I think, that in it there was nothing of the nature of tuberculosis.

### Trauma of the Hip simulating Coxa Vara.

Coxa vara, with its well-defined deformity of the neck of the femur, does not come within the province of this communication. Trauma of the soft parts around the hip may, however, give rise to signs which simulate coxa vara, and in these cases the only course to pursue, apart from the definite information afforded by an *x*-ray examination, is to watch the progress of the ailment.

The following two cases are examples of the condition:—

In the end of August 1905 a boy, aged twelve years, was brought to the out-patient department of the Western Infirmary with a complaint of lameness. Three weeks previously he had fallen on his right hip. On examination I found that he was distinctly lame and there was outward rotation of the limb. On investigating the mobility of the hip-joint it was observed that when passive flexion of the thigh on the abdomen was made the movement was accompanied by abduction, as is seen in coxa vara. He returned one week later with all his symptoms gone.

Some time before this I had seen a similar case in the out-patient department of the Royal Hospital for Sick Children, and so convinced was I then of the condition being due to curvature of the neck of the femur that I wrote to the patient's doctor (Dr. Lithgow, of Cleland) to that effect. I was agreeably surprised in the course of a few days to receive a letter from Dr. Lithgow informing me that the child had made a complete recovery.

These cases interested me as they showed me that a bruise of the hip may readily be mistaken for a condition of more serious import. The curious position which the limb assumes when flexion of the hip is made is more than a mere abduction of the thigh. There is associated with it external rotation, and the result is that the leg and foot come to lie across the sound knee, recalling to mind the initial step of the "Highland fling." If the case be one of coxa vara, elevation of the trochanter will generally be found, with accompanying shortening of the limb, and there will in all probability be a history of lameness of some duration, sometimes preceded by injury to the region. In the cases under consideration we have a history of recent injury, there is no evidence by measurement of any alteration in the relationship of the bony points, and, as in the case of acute synovitis, the condition is comparatively rapidly recovered from under expectant treatment.

If we look for a cause for the peculiar attitude adopted in flexion we are, I think, driven to the conclusion that as a result of the injury there has occurred effusion into the substance of the gluteus maximus, and it is to relieve tension in the infiltrated muscle during flexion of the hip that abduction and external rotation take place. My reason for presenting these cases is that I am not aware that attention has so far been drawn to the condition which they illustrate.

### Unrecognised Fracture of the Neck of the Femur.

In the fuller knowledge gained by the use of *x*-rays of the results of injury to bones, fracture of the neck of the femur in childhood is now known to occur with more frequency than was formerly believed to be the case. The peculiar difficulty attending the recognition of this fracture in the child arises from the fact that it may be unaccompanied by the marked disability which in the adult is the rule. I mention the following case as an illustration:—

On April 27 of this year a boy, aged eight years, was brought to me at the out-patient department of the Royal Hospital for Sick Children with a complaint of lameness of the left lower limb of from one to two months' duration, and of pain in the knee if he walked much. On examination, the usual signs of morbus coxæ (attitude, rigidity of the joint, etc.) were absent; but there was nearly 1½ inch of actual shortening. Examination under an anæsthetic did not throw any further light on the case. An *x*-ray plate was taken of both hip-joints and showed at once that there had been a fracture through the middle of the neck of the left femur, with upward displacement of the lower fragment.

Unfortunately there was not much chance of improvement of the boy's condition so far as lameness was concerned, but there was some comfort in being able to tell his friends that he was not suffering from hip-joint disease.

In conclusion, I would observe that while morbus coxæ bulks largely on our horizon, and properly so, the above cases show that there may be present in the region of the hip other conditions which demand recognition and careful treatment. It is, by attention to details and absence of precipitation, possible in many cases to recognise these conditions and so avoid errors in diagnosis and in the conduct of the case.

